# Pregnancy and Delivery in Patients with Mastocytosis Treated at the Polish Center of the European Competence Network on Mastocytosis (ECNM)

**DOI:** 10.1371/journal.pone.0146924

**Published:** 2016-01-21

**Authors:** Katarzyna Ciach, Marek Niedoszytko, Anna Abacjew-Chmylko, Izabela Pabin, Przemyslaw Adamski, Katarzyna Leszczynska, Krzysztof Preis, Hanna Olszewska, Dariusz G. Wydra, Rita Hansdorfer-Korzon

**Affiliations:** 1 Department of Obstetrics, Medical University of Gdansk, Gdansk, Poland; 2 Department of Allergology, Medical University of Gdansk, Gdansk, Poland; 3 Department of Gynecology, Gynecological Oncology and Gynecological Endocrinology, Medical University of Gdansk, Gdansk, Poland; 4 Department of Physiotherapy, Medical University of Gdansk, Gdansk, Poland; University of Bari Medical School, ITALY

## Abstract

**Objective:**

To present current guidelines regarding treatment of mastocytosis in pregnancy on the example of observed patients.

**Design:**

Case control national study.

**Setting:**

Polish Center of the European Competence Network on Mastocytosis (ECNM).

**Population or Sample:**

23 singleton spontaneous pregnancies in 17 women diagnosed with mastocytosis in years 1999–2014, before becoming pregnant.

**Methods:**

Prospective analysis outcomes of pregnancies and deliveries.

**Main Outcome Measures:**

Survey developed in cooperation with the Spanish Instituto de Estudios de Mastocitosis de Castilla-La Mancha (CLMast), Hospital Virgen del Valle, Toledo, Red Espańola de Mastocitosis (REMA), Spain.

**Results:**

All 23 pregnancies resulted from natural conception. Obstetrical complications recorded in the first trimester included spontaneous miscarriage (5 pregnancies). Four patients delivered preterm, including one delivery due to preeclampsia at 26 weeks which resulted with neonate death due to extreme prematurity. Five women delivered via cesarean: four due to obstetrical indications and one due to mastocytosis, during which no anesthesia related complications were recorded. Of patients delivering vaginally, two received extradural anesthesia, three required oxytocin infusion due to uterine hypotonia. No labor complications were recorded. In one woman with pregnancy-induced hypertension, early puerperium was complicated by the presence of persistent arterial hypertension. One neonate was born with the signs of cutaneous mastocytosis. Another neonate was diagnosed with Patau syndrome. Four women were treated for mastocytosis prior to conception and continued therapy after becoming pregnant. One patient was put on medications in the first trimester due to worsening of her symptoms. Pregnancy exerted only a slight effect on the intensity and frequency of mastocytosis-related symptoms observed. Worsening of the disease-related symptoms was documented in only four patients (23%). None of the patients showed the signs of anaphylaxis, either before becoming pregnant, or during pregnancy and puerperium.

**Conclusions:**

There is no contraindication to pregnancy when mastocystosis-related pathologies are under appropriate medical control.

## Introduction

Mastocytosis is a myeloproliferative disorder resulting from abnormal proliferation of mast cells (MCs) and resultant infiltration of various organs, especially bone marrow, skin, liver, spleen and lymph nodes. The prevalence of mastocytosis estimated at 13 per 100 000 in all age categories [1]. Classification proposed by WHO [2] includes various forms of mastocytosis, such as cutaneous mastocytosis and systemic forms (indolent, aggressive, mast cell leukemia, mast cell sarcoma, mastocytosis associated with non-mast cell associated hematological disease). While systemic mastocytosis is the most common type of this disease in adults, cutaneous form is typical for pediatric patients [2, 3].

**Fig 1:**
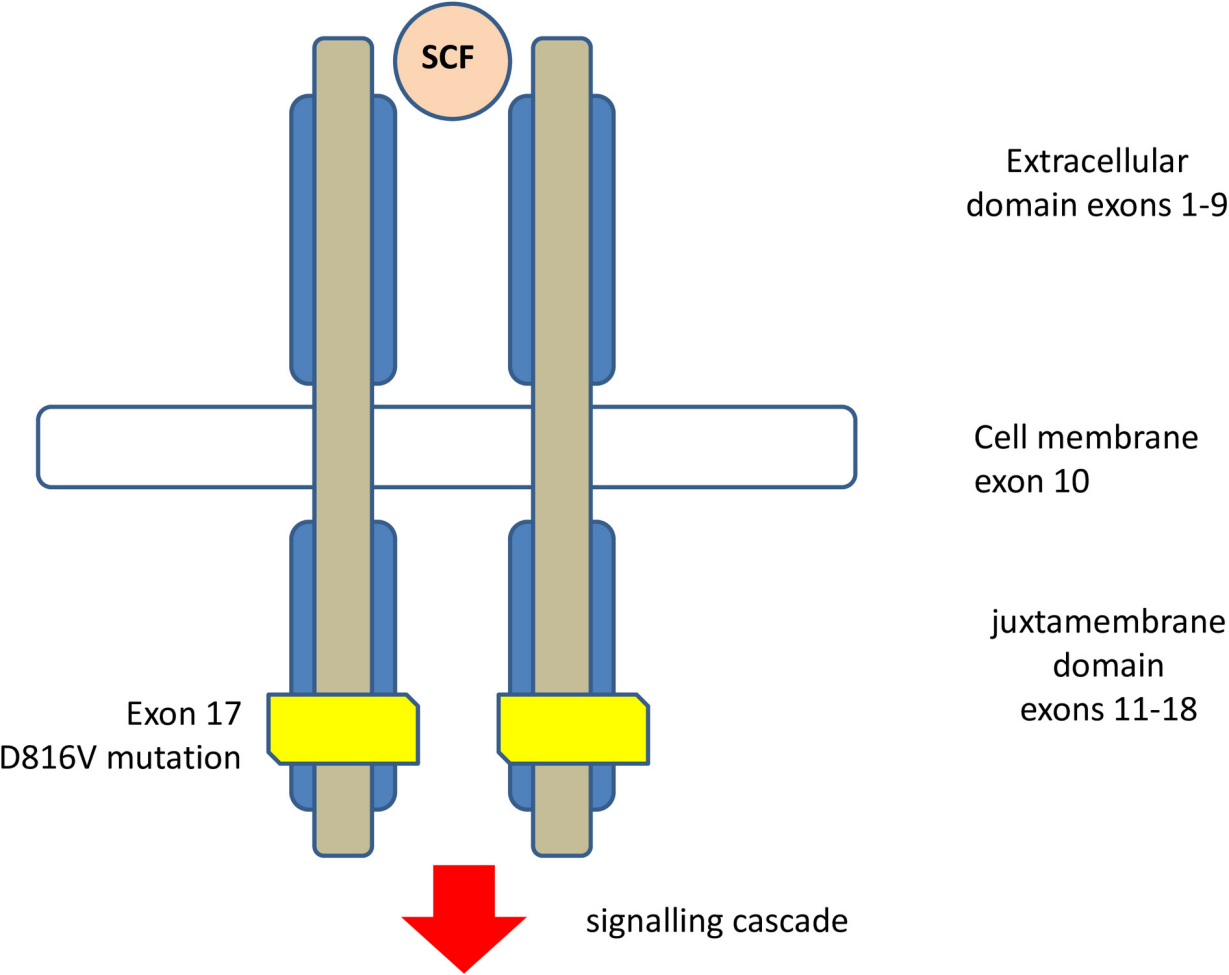
The D816V mutation in the catalytic domain of the c-Kit receptor downstream tyrosine kinase in peripheral blood mononuclear cells (by M. Niedoszytko).

Somatic activating mutations of the proto-oncogene KIT are the most common abnormalities in mastocytosis. KIT oncogene encodes a transmembrane protein KIT (CD117), which is a type III receptor tyrosine kinase [2, 4–6]. It is expressed by MC, hematopoietic progenitor cells of Cajal in the gastrointestinal tract. The interaction between KIT and its ligand, stem cell factor, plays a central role in regulating proliferation, growth, differentiation, adhesion, chemotaxis, and survival of MCs. A KIT D816V mutation has been found in approximately 90% of patients with systemic mastocytosis (SM), irrespective of WHO SM subtype [7–10]. The graph of the D816V mutation in the catalytic domain of the c-Kit receptor downstream tyrosine kinase in peripheral blood mononuclear cells is presented in Fig 1.

Symptomatology of mastocytosis is quite heterogeneous as MCs may infiltrate various organs. Most patients present with involvement of the skin, and more than half of them suffer from recurrent anaphylactic reactions including severe (near fatal or even fatal) cases. Common complaints include gastrointestinal manifestations, osteopenia or osteoporosis, depression and neurological symptoms. Aggressive mastocytosis is a rare condition requiring cytoreductive treatment due to impairment of affected organs, e.g. cytopenia, liver and spleen impairment, weight loss and malabsorption.

The most prominent symptoms of mastocytosis, i.e. flush, hypotension and anaphylactic reactions, are associated with the release of MC mediators. The most common triggers for the reaction are insect stings, drugs (including NSAIDs, anesthetics and antibiotics), foods and mechanical stimulation. The list of drugs that can be safely administered to patients with mastocytosis can be found on the web page of the European Competence Network on Mastocytosis [11].

Mastocytosis may be also diagnosed in pregnant women which raises questions about both maternal and child safety, treatments that can be used in pregnancy, delivery and breastfeeding.

Pregnancy represents a unique challenge for maternal organism. Tremendous endocrine and immunological modifications that occur as an adaptation to implanted embryo ensure a successful outcome of pregnancy. These necessary changes in hormonal status and a shift towards anti-inflammation may, however, cause a dysregulation in the number of MCs and their behavior. MCs have been shown to exhibit beneficial effect in pregnancy, contributing to implantation, placentation and fetal growth due to release of glycan-binding protein galectin-1, and as such, are critically implied in the fetomaternal interface [9]. MCs express estrogen and progesterone receptors and therefore are present in the myometrium and placenta. The number of MCs in the myometrium increases with pregnancy, and seems to affect the second stage of labor [12–15]. Elevated levels of histamine have been reported to increase pregnant myometrial contractions *in vitro*, which may be associated with an increase in preterm labor *in vivo* [16]. Mastocytosis is perceived as a medical management dilemma due to its potential for unpredictably heightened MC activity in response to various physiologic states, including pregnancy [17–19]. Also the stress related to labor, delivery and medications has potential to activate the disease. Therefore, pregnancy may be associated with potential complications of mastocytosis.

The aim of this paper is to present current guidelines regarding treatment of mastocytosis in pregnancy on the example of 17 patients from the Polish Center of the European Competence Network on Mastocytosis.

## Materials and Methods

Our prospective analysis included the outcomes of pregnancies and deliveries in 17 patients who have been diagnosed with mastocytosis in 1999–2014, before becoming pregnant. All the patients were qualified for treatment at the Polish Center of the European Competence Network on Mastocytosis (ECNM).

The patients completed a survey developed in cooperation with the Spanish Instituto de Estudios de Mastocitosis de Castilla-La Mancha (CLMast), Hospital Virgen del Valle, Toledo, Red Espańola de Mastocitosis (REMA), Spain [20].

The analyzed group included 10 women with indolent systemic mastocytosis (ISM) and 7 patients with cutaneous mastocytosis. One patient with ISM was recently diagnosed with aggressive systemic mastocytosis (ASM) and is now during aggressive chemiotherapy (cladribine).

All participants gave their informed consent to participate in this combined, prospective and retrospective study.

Seventeen women were pregnant 23 times: once (n = 12), twice (n = 4) or three times (n = 1). All 23 pregnancies were singleton pregnancies. Fifteen women delivered, 13 once and 2 twice. Five patients had a total of 6 miscarriages, all in the first trimester.

Diagnosis of mastocytosis was established upon referral to the ENCM, on the basis of skin and bone marrow (BM) biopsy findings, in line with the World Health Organization (WHO) criteria [21]. At the time of diagnosis the following parameters of mastocytosis were determined: percentage of atypical MCs in bone marrow smear, serum tryptase levels (ng/ml), detection of c-kit somatic mutations, including KIT D816V, CD25 and CD2 expression.

Median age at pregnancy was 30 years (range 21–38 years).

The protocol of the study was approved by the Local Bioethics Committee at the Medical University of Gdansk. A written informed consent was given by participants for their clinical records to be used in this study. Additionally, patient data was anonymized prior to analysis.

Clinical data were extracted from medical histories of the patients or obtained with a survey. The occurrence of symptoms associated with the release of MC mediators, such as pruritus, flushing, skin lesions, abdominal cramps, gastrointestinal symptoms (dyspepsia, malabsorption), musculoskeletal pain, fatigue, diarrhea and anaphylaxis, was analyzed for three periods: pregestation, pregnancy (first, second and third trimester), and puerperium.

All symptoms were graded according to the ECNM frequency criteria: (1) absent, (2) daily, (3) weekly (on > 1 but < 7 days per week), (4) monthly, and (5) occasionally (< once a month). Any changes in the occurrence of symptoms during pregnancy and puerperium were recorded. Worsening of the symptoms was defined as an evident increase in their frequency and/or development of a new, previously absent symptom (during any trimester). Additionally, the course of pregnancies were analyzed. Pregnancies both before and after the onset of mastocytosis were registered. In case of miscarriages no histopathological analysis was performed.

## Results

Sixteen patients conceived normally, and one experienced some problems due to hyperprolactinemia. All 23 pregnancies resulted from natural conception. The course of pregnancy during mastocytosis in 17 patients are presented in Table 1.

**Table 1:** The course of pregnancies and deliveries in 17 patients with mastocytosis.

Patient no.	Type of disease	Year of diagnosis	Onset of symptoms	Onset of symptoms	Age during pregnancy	delivery (D)/ miscarriage (M)	Pregnancies before mastoc.	Drugs before pregnancy	Drugs during pregnancy	Drugs after pregnancy	Complications during pregnancy	Week of delivery	Type of delivery	Anesthesia during labour	Oxytocin
1	ISM + UP	2005	1995	2012	27	D	0	0	Ce, L,R, mP, P, MTX, alpha methyldopa, metoprolol succinate, human albumin; during labour—Ce, R, P; Cl, hydrocortisone acetate, urapidil hydrochloride	Ce, P, R, alpha methyldopa, urapidil hydrochloride, verapamile hydrochloride, diazepam, nadroparine calcium, enalapril maleate	pregnancy induced hypertension, preeclampsia, hypoalbuminemia, extreme preterm delivery (26^th^ week)	26	CS	spinal	0
2	ISM	1999	1999	1990	21	D	0	0	0	0	preterm delivery	36	V	0	0
3^a^	ISM	2005	1990	2007	30	D	0	Ce, R	Ce, R	Ce, R	0	41	V	epidural	0
3^b^				2008	31	D		0	0	0	0	38	V	0	0
4	CM	2006	1999	2010	25	D	0	Ce, R	Ce, R, P, H	Ce, R	severe deep vein thrombosis, preterm labor, LBW	38	V	0	0
5	ISM	2005	1993	1989	24	D	0	0	0	0	0	40	V	0	0
6^a^	CM	2008	2008	2012	30	D	0	0	0	0	0	40	V	0	1
6^b^				2010	28	M		0	0						
7	ISM + UP	2012	2008	2014	30	D	0	0	0	0	0	39	CS	spinal	0
8	CM	2009	2009	2012	31	D	0	0	Rovamycine	0	Toxoplasmosis	42	V	epidural	1
9	CM	2014	1995	2008	27	D	0	0	0	0	preterm delivery	36	CS	spinal	0
10^a^	ISM	2014	2003	2011	33	D	1 D (2002)	0	0	0	preterm delivery,LBW (Patau syndrome)	37	V	0	0
10^b^				2014	36	M		0	0						
11^a^	ISM	2006	2006	2009	21	D	0	Ce	0	0	0	38	V	0	0
11^b^				2014	26	D		Ce	0	0	0	41	V	0	0
12	ISM	2012	1996	2001	34	D	3D (1990, 1992, 1993)	0	0	0	0	41	CS	spinal	0
13^a^	ASM	2010	2008	2011	34	D	1 D (2003)	dL	dL	dL	0	40	V	0	1
13^b^				2008	31	M		0	0	0					
13^c^				2009	32	M		0	0	0					
14	CM	2010	2010	2015	31	M	0	0	0	0					
15	ISM	2012	1998	2014	34	M	0	0	0	0					
16	CM	2010	2005	2010	36	D	0	0	0	0	0	40	CS	spinal	0
17	CM	2013	2005	2014	38	D	2 D (1995, 1997)	0	0	0	vaginal bleeding in the first trimester and preterm labor without delivery	40	V	0	0

a—first pregnancy

b–second pregnancy

c–third pregnancy

0 –no, 1- yes; D–delivery, M–miscarriage; ISM–indolent systemic mastocytosis, ASM–aggressive systemic mastocytosis, CM–cutaneous mastocytosis, UP—urticaria pigmentosa; CS—cesarean section, V–vaginal, LBW—low birth weight, Ce—cetirizine dihydrochloride, L–loratadine, dL–desloratidine, Cl—clemastine fumarate, MTX–methotrexate, P–prednisone, mP–methylprednisolone, R- ranitidine, H—Low molecular weight heparin

Obstetrical complications recorded in the first trimester included spontaneous miscarriage (n = 5, 29.4%) (no. 6a, 10a, 13bc, 14 and 15). One patient with ISM who developed ASM after the recorded pregnancies (no. 13b and 13c) had miscarried twice, before she eventually delivered a baby (no. 13a). Before the onset of mastocytosis she had a term delivery.

Thirteen women delivered once, and another two twice. Overall, 17 pregnant women with mastocytosis delivered a total of 17 neonates. All 17 neonates were born between 26 and 42 weeks of gestation, at 38.41 weeks on average.

Five women delivered via cesarean: four due to obstetrical indications (lack of labor progression, preeclampsia, transverse position of the fetus, fetopelvic disproportion) and one (no. 7) due to mastocytosis. All 5 patients who delivered via cesarean received subarachnoid anesthesia; no anesthesia-related complications were recorded. Twelve patients delivered vaginally. Two of them received extradural anesthesia, and three required oxytocin infusion due to uterine hypotonia. No labor complications were recorded.

Four patients (no. 1, 4, 8 and 17) experienced obstetrical complications in the second trimester (Table 1). In one woman with pregnancy-induced hypertension (no. 1), early puerperium was complicated by the presence of persistent arterial hypertension and limb edema. The patient was administered prednisone, ranitidine hydrochloride, alpha methyldopa, urapidil hydrochloride, verapamil hydrochloride, diazepam, nadroparin calcium, cetirizine dihydrochloride and enalapril maleate. Due to poor status of the neonate, lactation was inhibited. On postoperative day 5, the patient was discharged home on her request, in good general status, with arterial pressure between 150/80 mmHg and 170/120 mm Hg and persistent lower extremity edema. A protein-rich diet was prescribed due to persistently low serum level of albumins (25 g/l) [22]. The other cases experienced severe deep thrombosis (no. 4), toxoplasmosis (no. 8), vaginal bleeding in the first trimester and preterm labor without delivery (no. 17).

Four patients delivered preterm (at 26–37 weeks of gestation), including one delivery due to preeclampsia at 26 weeks (no. 1). The other preterm deliveries occurred in the 36^th^ (no. 2, 9) and 37^th^ weeks (no. 10a). Twelve women delivered at term. Birthweight of the 17 neonates ranged between 580 g and 4340 g (mean 3291 g). Three neonates had a low birth weight. The baby born at 26 weeks of gestation (580 g) died at 22 days of life due to extreme prematurity, circulatory failure, grade 3 intraventricular hemorrhage, kidney failure, cardiopulmonary failure (no. 1). Another neonate (no. 10a) was diagnosed with Patau syndrome (1790 g). The third neonate had weight restriction without any other complications (no. 4).

One neonate was born with the signs of cutaneous mastocytosis (no. 16).

Four women (no. 3a, 4, 11ab and 13a) were treated for mastocytosis prior to conception and continued therapy after becoming pregnant. Another patient (no. 1) was put on medications in the first trimester due to worsening of her symptoms.

Seven patients (no. 1, 4, 7, 9, 10a, 13a and 16) complained on pruritus before becoming pregnant, as well as in pregnancy and puerperium. Patients no. 7, 10a and 13 presented with mild, patients no. 4,9 and 16 with moderately severe, and patient no. 1 with extremely severe pruritus. Seven women (no. 1, 4, 7, 9, 10a, 13a and 16) presented with mild (no. 4, 7, 9, 13 and 16), moderately severe (no. 10a) or extremely severe urticaria (no. 1) prior to conception, in pregnancy and puerperium. Patients no. 1, 4, 10a and 13a reported daily occurrence of pruritus and urticaria during the course of pregnancy and puerperium. Seven patients presented with mild (no. 7 and 16), moderately severe (no. 4, 9, 10a and 13a) or extremely severe skin lesions (no. 1) before becoming pregnant, as well as during pregnancy and puerperium. Five women (no. 1, 7, 9, 10a and 16) reported sporadic occurrence of mild abdominal cramps, dyspepsia, malabsorption and diarrhea prior to conception, in pregnancy and puerperium. These complaints were absent in the remaining women. None of the patients showed the signs of anaphylaxis, either before becoming pregnant, or during pregnancy and puerperium. Patients no. 7, 9 and 16 complained on sporadic occurrence of mild-degree fatigue and musculoskeletal pain prior to conception, in pregnancy and puerperium.

Pregnancy exerted only a slight effect on the intensity and frequency of mastocytosis-related symptoms observed in our 17 patients. Worsening of the disease-related symptoms was documented in only four patients (23%, no. 1, 4, 10a and 9). Specifically:

1. One patient (5.9%, no. 1) showed evident exacerbation of pruritus, urticaria and skin lesions as compared to her pregestational status.
2. One patient (no. 4) showed exacerbation of urticaria, and another one (no. 10a) exacerbation of skin changes in the second and third trimester. The intensity and frequency of MC mediator-related symptoms in the remaining 88% of patients were unchanged.
3. Two patients with systemic mastocytosis (no. 1 and 10a) complained on exacerbation of fatigue during pregnancy as compared to pregestational period.
4. Patient no. 9 reported greater intensity and frequency of musculoskeletal pain than before pregnancy.
5. In four patients (no. 7, 13a, 16 and 17), the intensity and incidence of skin symptoms (pruritus, urticaria) in puerperium were significantly higher than prior to conception and during pregnancy.

The course of pregnancies correlated with chosen parameters of mastocytosis: percentage of atypical MCs in bone marrow smear (mean 2,95% ± 5,7), serum tryptase levels (mean 27,7 ± 6,3 ng/ml), detection of c-kit somatic mutations (26,7%) and KIT D816V mutation (6,7%), CD25 (94,1%) and CD2 (94,1%) expression, are presented in Table 2.

**Table 2:** Chosen parameters of the disease and outcomes of pregnancies in patients with mastocytosis.

Patient no.	Type of disease	Deliveries	Miscarriages	atypical MCs in bone marrow smear (%)	Serum tryptase (ng/ml)	c-kit mutation	CD2 expression	CD25 expression	Involvement of other organs	Skin involvement	Urticaria pigmentosa
1	ISM	1	0	9	30,2	0	1	1	0	1	0
2	ISM	1	0		24,5		1	1	0	0	1
3	ISM	2	0	11,9	77,8	1*	1	1	0	1	1
4	CM	1	0	1	8,84	0	0	0	0	0	1
5	ISM	1	0	0,8	26,3	0	1	1	0	1	1
6	CM	1	1	0	7,58		1	1	0	1	1
7	CM	1	0	0	12,4	0	1	1	0	1	1
8	CM	1	0	0	6,3	0	1	1	1	1	1
9	CM	1	0	0	6,3	0	1	1	1	1	1
10	ISM	1	1		41,4	1	1	1	1	1	1
11	ISM	2	0	0,3	14,5	0	1	1	1	1	1
12	ISM	1	0	0	35,4	1	1	1	1	1	1
13	ISM	1	2	19	173	1	1	1	1	1	1
14	CM	0	1	0	11	0	1	1	1	1	1
15	ISM	0	1	2,3	16	1	1	1	1	1	1
16	CM	1	0	0	6,3	0	1	1	1	1	1
17	CM	1	0	0	7,58	0	1	1	1	1	1

ISM–indolent systemic mastocytosis, CM–cutaneous mastocytosi s; 0 –no, 1 –yes

*—presence of D816V mutation

## Discussion

Available data on the outcomes of pregnancy and labor in women with mastocytosis originate from single case reports [16, 22–30] and a few case series [20, 31, 32]. Overall, the data of 60 pregnant women with mastocytosis have been published so far. The outcome of the pregnancies and the influence of gestation on the symptoms of mastocytosis have been summarized in Table 3.

**Table 3:** The course of pregnancy in patients with mastocytosis–comparison of own study and published research.

Feature, n (%)	Women	Pregnancies	Deliveries	Type of mastocytosis	Problems with conception	Miscarriage	Intrapartum complications	Complications during pregnancy	Symptoms in Pregnancy Worse	Symptoms in Pregnancy Constant	Symptoms in Pregnancy Improve	Symptoms After pregnancy Worse	Children with mastocytosis
Worobec [32]	8	13	11	4 CM, 3 ISM	3 (37,5)	2 (25)	1 acceleration of fetal heart rate during labor, 1 excessive bleeding after delivery	1 preeclampsia. 3 LBW, 1 hydrocephalus and developmental delay	5 (62,5)	3* (37,5)	*	4 (50)	0
Burns [31]	12	12	12	NA	NA	NA	0	0	4 (33,3)	7 (58,3)	1 (8,3)	NA	NA
Matito [20]	30	45	45	1 CM, 25 ISM, 1 WDSM	1 (3,3)	9 (30)	5 MC-mediator release symptoms (anaphylaxis)	3 preterm delivery, 4 LBW, 2 neonatal respiratory distress	10 (22)	20 (45)	15 (33)	9 (10)	1 CM
Maatouk [30]	3	3	3	NA	NA	NA	NA	NA	NA	NA	NA	NA	NA
Ciach	17	22	17	8 CM, 9 ISM	2 (10,8)	5(29,4)	0	1 preeclampsia, preterm delivery, LBW, 1 preterm labor, 2 preterm delivery, 1 deep vein thrombosis, preterm labor, LBW, 1 toxoplasmosis, 1 LBW (Patau syndrome)	4 (23,5)	13 (4,1)	0	4 (23,5)	1
Donahue [16]‡	1	4	2	1 CM	0	2	0	1 preeclampsia, preterm delivery, 1 anaphylactic reaction, preterm labor and delivery	2	0	0	0	0
Kehoe [23]‡	1	1	1	1 ISM	0	0	0	0	0	0	0	0	0
Madendag [24]‡	1	1	1	1 CM	0	0	0	1 preterm labor	1	0	0	0	0
Watson [25]‡	1	2	2	1 ISM	0	0	1 anaphylactic reaction	2 preterm delivery, 1 anaphylactic reaction	0	2	0	0	0
Villeneuve [26]‡	1	1	1	1 CM	0	0	0	0	0	0	0	0	0
Garcia [27]‡	1	1	1	1 ISM	NA	NA	NA	NA	NA	NA	NA	NA	NA
Gupta [28]‡	1	1	1	1 CM	0	0	0	0	0	0	0	0	0

* together no change and improved

‡ case resports did not allow to present a percentage value of the parameters

ISM–indolent systemic mastocytosis, CM–cutaneous mastocytosis WDSM—well-differentiated systemic mastocytosis; LBW—low birth weight; NA not available

The presented results show that women with mastocytosis are fertile and can become pregnant and deliver providing adequate control of MC-mediated symptoms. Worobec et al. published a retrospective analysis of 169 female patients, who were treated for mastocytosis. From 9 patients in the reproductive age, 8 conceived after being diagnosed with mastocytosis and delivered a total of 11 babies [32].

As described in the literature, difficulties in getting pregnant were not frequently recorded. In our study no patients were treated actively for infertility; one conceived on medication for hyperprolactinemia, another had an early miscarriage. In the study of Worobec et al. only one patient (12,5%) was treated with clomiphene [32]. Matito et al. in a study of the Spanish Network on Mastocytosis (REMA) reported that 1 pregnancy (2%) was achieved by in vitro fertilization due to a history of infertility [20].

According to literature, between 25% and 30% of pregnant women with mastocytosis may experience spontaneous miscarriages [20, 32]. Our study results are consistent with this data as 29.4% women had a miscarriage.

The absence of severe maternal and infantile complications suggests that patients with nonaggressive form of mastocytosis should not be advised against pregnancy. In the study published by Bruns and Hartmann [31] none of the 12 patients experienced significant complications during labor and delivery. However, clinicians should be aware of the risk of preterm labor in patients with this disease. Donahue et al. showed a case of preterm labor and delivery in two consecutive pregnancies that were a consequence of this condition [16]. Also Matito et al. [20] and authors of case reports [24, 25] report an increased frequency of preterm deliveries. In our study two patients had symptoms of preterm labor (delivered at term), three delivered at the 36–37^th^ week and one had a extreme preterm delivery at the 27^th^ week due to preeclampsia. All these cases especially the last could be a consequence of mastocytosis.

The review of the literature (Table 3) suggests that in patients with mastocytosis during pregnancy, there is an increased risk of complications caused by the activation of coagulation mechanisms. Including our paper, three cases of heavy preeclampsia, that lead to preterm deliveries, have been reported. Additionally, one episode of deep thrombosis was described in our group. On the other hand, although several patients with mastocytosis who have been treated at the Polish Center of ECNM experienced thrombotic events, only one such episode was recorded in a pregnant woman (no. 4). In contrast to most patients with thrombotic events, who were eventually diagnosed with antiphospholipid syndrome, this woman with deep venous thrombosis did not show any abnormalities in hematological parameters.

As the majority of pregnant patients suffer from indolent systemic or cutaneous mastocytosis, the most important problem which needs to be addressed is the risk of anaphylactic reaction. Theoretically, hormonal changes associated with pregnancy and puerperium may promote anaphylactic reactions. In our study there was no patient with an anaphylactic reaction during the pregnancy, labor or puerperium. Matito et al. [20] reported 5 patients (17%) who suffered anaphylactic episodes during pregnancy– 2 cases were idiopathic and 3 cases were triggered by external factors (a hymenoptera sting, kiwifruit ingestion and codeine). Donahue et al. [16] and Watson et al. [25] reported 2 additional cases (idiopathic and due to allergy to terbutaline) that lead to preterm delivery.

During the process of labor anaphylactoid reactions may triggered by physical stimuli or drugs administered during delivery, especially anesthesia. In line with the ECNM [2] and American College of Radiology guidelines [33], premedication was advised prior to delivery in all patients included in our series. Such approach was shown to be safe and free from any side effects. Although none of our patients had a history of hypersensitivity to local anesthetics, it should be remembered that anaphylactic reaction can be also triggered by other factors (e.g. allergens, exercise, stress) or combinations thereof [34]. Researchers from REMA group [20] used various combinations of antihistamines and corticosteroids in 38% of their patients and still MC-mediator release symptoms during or immediately after labor were observed in 5 cases. Such combined treatment was not administered to any of patients included in Worobec’s cohort [20]. Villenneuve et al. reported the case of a pregnant woman with mastocytosis who showed the signs of intolerance to local anesthetics before becoming pregnant [26]. Skin prick tests facilitated selection of a safe anesthetic agent (lignocaine), and the patient eventually delivered via cesarean under subarachnoid anesthesia. Testing for drug hypersensitivity is generally recommended only in patients with a history of drug allergy [4]. Medications, such as glucocorticoids, antihistamines and epinephrine, should be also available during the critical phases of labor and in early postpartum period [35].

According to literature [20, 32], between 20% and one third of pregnant women with mastocytosis may experience worsening of the disease-related symptoms. Our study results are consistent with this data, as we observed exacerbation in 24% of pregnant patients with mastocytosis.

Worobec et al. published that in 5 women, the symptoms of mastocytosis worsened during pregnancy. Bruns and Hartmann [31] evaluated that during pregnancy, four patients experienced deterioration of mastocytosis symptoms, seven reported no change, and one had improved. Matito et al. published that approximately 22% of pregnant women experienced clinical worsening, 30% improvement during the first trimester, and another 50% did not show any changes in MC mediator-related symptoms [20]. Interestingly, the symptoms typically worsened in the first or third trimester [20], when Th1-mediated pro-inflammatory conditions dominate [9]. Although women diagnosed with mastocytosis are often required to continue treatment (including antihistamines) during pregnancy, usually the dosage is decreased due to fetal safety concerns [32]. This, as well as an irregular medication intake, may contribute to the worsening of mastocytosis symptoms. However, the exacerbation of symptoms in pregnancy is not a universal phenomenon, as the vast majority of our pregnant patients did not experience any worsening.

In our study we made an attempt to correlate the course of pregnancies and the levels of chosen parameters of mastocytosis and it was partly observed. The highest levels of atypical MCs in bone marrow smear (19%) and of serum tryptase (173 ug/ml) were noted in patient no. 13, who developed an aggressive form of systemic mastocystosis a few years after her last pregnancy. She had two unsuccessful attempts of having a baby after the diagnosis of mastocytosis, as she miscarried two pregnancies. What is interesting, five years before the onset of symptoms and then three years after the diagnosis, she had successful deliveries at term. Apart from this situation, miscarriages in other patients were observed with low values of the mentioned parameters. Moreover, an severe complication of pregnancy–pregnancy induced hypertension with preeclampsia causing extreme preterm delivery—was observed in patient no. 1, who did not have high levels of atypical MCs in bone marrow smear (9%) and of serum tryptase (30,2 ug/ml). The data on this subject is limited as none of the cited publications [20, 16, 31, 32] had analyzed such parameters before. However, our data suggests that low levels of these chosen parameters allow for conception and successful pregnancy.

Further analysis showed that women who had a mutation in the c-kit receptor had a higher percentage of atypical MCs in bone marrow smear and level of serum tryptase, but did not have a more severe course of disease than the rest of the women. This was also noted by Worobec et al. [32].

Administration of antihistamines to pregnant women with mild mastocytosis raises many controversies. Although second-generation antihistamines (sgAHs) are widely used in the treatment of allergic diseases, only loratadine, and perhaps also desloratidine [36], can be administered to pregnant patients with mastocytosis due to lack of sufficient safety data. While none of the previous studies provided any evidence for a teratogenicity of sgAHs, some first generation antihistamines were shown to exert unfavorable effect on fetuses during the course of animal studies [37, 38]. Three pregnant women from our series used antihistamines (cetirizine, desloratidine) in pregnancy and none of them showed any adverse effects.

Importantly, only one neonate included in our series presented with the signs of mastocytosis. Familial cutaneous mastocytosis is a rare condition that has been reported in several cases so far, including one family treated at our center [6]. Also researchers from REMA group found familial mastocytosis in only 1 out of the 45 children included in their series (several years after birth) [20]. Most patients with familial mastocytosis present with a benign cutaneous form of the disease and atypical mutation of the *KIT* gene [6].

## Conclusion

Despite our current understanding of mastocytosis, still little is known on the influence of pregnancy on clinical features, tolerance to medications and outcomes of pregnant patients with this condition. In general, there is no contraindication to pregnancy when MC-related pathologies are under appropriate medical control. Undiagnosed and not appropriately treated mastocytosis may lead to severe pregnancy complications, including fetal demise [25]. Women who were diagnosed with MC-mediated or -associated disorders, especially those whose disease is active, should be carefully advised by medical specialists to avoid severe pregnancy complications and to monitor progression of the disease.
